# Physical Activity Surveillance in Parks Using Direct Observation

**DOI:** 10.5888/pcd11.130147

**Published:** 2014-01-02

**Authors:** Phillip Ward, Thomas L. McKenzie, Deborah Cohen, Kelly R. Evenson, Daniela Golinelli, Amy Hillier, Sandra C. Lapham, Stephanie Williamson

**Affiliations:** Author Affiliations: Thomas L. McKenzie, San Diego State University, School of Exercise and Nutritional Sciences, San Diego, California; Deborah Cohen, Daniela Golinelli, Stephanie Williamson, RAND Corporation, Santa Monica California; Kelly R. Evenson, University of North Carolina at Chapel Hill, Chapel Hill, North Carolina; Amy Hillier, University of Pennsylvania, Philadelphia, Pennsylvania; Sandra C. Lapham, Behavioral Research Center of the Southwest/PIRE, Albuquerque, New Mexico.

## Abstract

**Introduction:**

Primary features of observational public health surveillance instruments are that they are valid, can reliably estimate physical activity behaviors, and are useful across diverse geographic settings and seasons by different users. Previous studies have reported the validity and reliability of Systematic Observation of Play and Recreation in Communities (SOPARC) to estimate park and user characteristics. The purpose of this investigation was to establish the use of SOPARC as a surveillance instrument and to situate the findings from the study in the context of the previous literature.

**Methods:**

We collected data by using SOPARC for more than 3 years in 4 locations: Philadelphia, Pennsylvania; Columbus, Ohio; Chapel Hill/Durham, North Carolina; and Albuquerque, New Mexico during spring, summer, and autumn.

**Results:**

We observed a total of 35,990 park users with an overall observer reliability of 94% (range, 85%–99%) conducted on 15% of the observations. We monitored the proportion of park users engaging in moderate-to-vigorous physical activity (MVPA) and found marginal differences in MVPA by both city and season. Park users visited parks significantly more on weekend days than weekdays and visitation rates tended to be lower during summer than spring.

**Conclusion:**

SOPARC is a highly reliable observation instrument that can be used to collect data across diverse geographic settings and seasons by different users and has potential as a surveillance system.

## Introduction

The surveillance of physical activity, an important determinant of health and longevity, has been limited to self-report until recently. The addition of accelerometry as part of the data collected in the 2004 National Health and Nutrition Examination Survey (NHANES) allowed, for the first time, an objective assessment of moderate-to-vigorous physical activity (MVPA) in a representative sample of Americans (1). The findings demonstrated a significant discrepancy between self-reports and objective measures. On the basis of self-report, approximately 50% of American adults appeared to meet the national physical activity guidelines, but according to accelerometry, fewer than 5% did (1,2).

Because people are biased in their reporting and most lack the capacity to accurately measure their own physical activity without the assistance of technology, methods other than self-report are necessary to study trends in physical activity (3,4). Given that physical inactivity is an underlying cause of cardiovascular disease, hypertension, and type 2 diabetes and is estimated to be responsible for 10.8% of all-cause mortality, including deaths from breast and colon cancer (5–7), increasing population physical activity is an important national public health goal (8). Thus, the ongoing investigation of the epidemiology of physical activity is a critical endeavor at both the local and national levels.

Improved surveillance tools are needed to understand the degree to which the public health goals for physical activity are being met. The NHANES accelerometry study follows too few people to generalize to local or state populations, and self-reports as measured through the Behavioral Risk Factor Surveillance System (BRFSS) or Youth Risk Behavior Surveillance System (YRBSS) do not appear to be sufficiently valid for measuring MVPA (2,8,9). An alternative approach is direct observation, in which physical activity can be observed as it occurs in key community locations. This type of surveillance is already being used for monitoring traffic patterns through the placement of motor vehicle counters in representative streets. Electronic devices have proven to be useful in measuring the number of users of trails and customers in stores, but they do not accurately distinguish important characteristics of individuals, such as their levels of physical activity, which are an important contributor to population health.

As work becomes more sedentary, physical activity occurs primarily during transportation and leisure activity. The most common form of transportation involving physical activity is walking; however, given the convenience and ubiquity of motorized transport, walking constitutes a small fraction of trips (10). Leisure-time activities are important contributors to MVPA and thus deserve monitoring. Leisure-time physical activity usually occurs at home, on public streets, in parks, and in private health clubs; of these locations, streets and parks are the most suitable for surveillance activities.

The System for Observing Play and Recreation in Communities (SOPARC) is a systematic, direct observation assessment tool that has been used across time, seasons, and geographic locations to compare patterns of physical activity in parks and park features (11). SOPARC has been the primary data collection tool in more than 40 peer-reviewed publications describing studies (12–19). SOPARC’s activity codes have been validated both by heart rate monitoring and energy expenditure measured via oxygen uptake (20,21). SOPARC data have also been demonstrated to reliably estimate total park use during daylight hours by using only three to four 1-hour observations per day during 3 to 4 days per week (22).

Studies using SOPARC have documented the characteristics of parks (accessibility, usability, and whether or not they provide supervision, equipment, and organized activities) and park users (levels and types of physical activity, sex, race/ethnicity, and age group) (12,13,15,18,20). These studies consistently report that most park users (>60%) are male and sedentary when observed. Seniors (adults aged >60 y) are typically the age group to use parks the least. The built features of parks such as playgrounds, sports fields, tennis and basketball courts, and, in particular, walking trails have been shown to affect physical activity across the life span (12–19). Parks in lower-income areas have been shown to have fewer built features (12–14,23,24) and fewer users (23,24) than those in higher-income areas. Most park studies have focused on a specific population within a small geographic area and have been of short duration. Few have reported the influence of seasonality on PA (19,25), and this is an understudied area.

To date, SOPARC has met 2 of 3 critical requirements of a public health surveillance instrument (26) in that it is valid (21,22) and it has reliably estimated total park use during daylight hours (26). However, none of the extant studies has assessed the usefulness of SOPARC as a surveillance instrument to measure park and user characteristics across diverse geographic settings and seasons by different users, which is a necessary feature of a public health surveillance instrument. The purpose of our investigation was to establish the usefulness of SOPARC as a surveillance instrument and to situate the findings from the study within the context of the literature.

## Methods

### Settings and park selection

To examine seasonal and geographic variability, we collected data over spring, summer, and autumn for 3 years in 4 locations within the United States: Philadelphia, Pennsylvania; Columbus, Ohio; Chapel Hill/Durham (hereafter called Chapel Hill), North Carolina; and Albuquerque, New Mexico. Institutional review boards in each site provided approval for the study, and in each city, 6 neighborhood parks were chosen on the basis of their facilities and size. Parks were excluded if they were to undergo construction during the study period or if they were considered too dangerous for project staff. Selected parks varied in size from a mean of 6.9 acres in Columbus to 13.5 acres in Chapel Hill (Table 1a, Table 1b). Eleven of the 24 parks had recreation centers with full-time programing staff; within cities, centers ranged from none in Albuquerque parks to all 6 in Philadelphia. In each city, we studied 2 to 4 parks in higher-income neighborhoods and 2 to 4 in lower-income neighborhoods.

**Table 1a:** Characteristics of Selected Parks by City

Park Characteristics	Chapel Hill, North Carolina	Albuquerque, New Mexico	Columbus, Ohio	Philadelphia, Pennsylvania
Mean acres (range)	13.5 (7–24)	7.3 (4–13)	6.9 (3.9–13)	6.8 (3.6–12)
No. of parks with recreation centers and full-time program staff	2/6	0/6	3/6	6/6
Population density (mean no. within .5-mile radius)	5,944	4,473	7,532	18,323
Households in poverty within .5-mile radius (%)	10.3	15.8	20.6	28.5
Households in poverty citywide (%)	8.8	11.2	14.8	24.3

**Table 1b:** Characteristics of Park Users by City

Park User Characteristics	N = 13,735,^a^ n (%)	N = 6,862,^a^ n (%)	N = 6,502,^a^ n (%)	N = 9,280,^a^ n (%)
**Sex**				
Male	7,265 (53)	3,398 (50)	3,382 (52)	5,633 (61)
Female	6,092 (47)	3,464 (50)	3,120 (48)	3,647 (39)
**Race/ethnicity**				
White	8,710 (65)	3,590 (53)	1,401 (22)	4,079 (44)
Black	1,682 (13)	179 (3)	4,307 (67)	3,624 (39)
Latino	1,167 (9)	2,538 (37)	296 (5)	630 (7)
Other	1,797 (13)	504 (7)	428 (7)	854 (9)
**Age, y**				
Children (<12)	4,164 (31)	2,188 (32)	3,208 (49)	3,030 (33)
Teens (>13–20)	830 (6)	687 (10)	831 (13)	1,513 (16)
Adults (> 21–59)	7,873 (59)	3,635 (53)	2,403 (37)	4,623 (50)
Seniors (>60)	490 (4)	352 (5)	60 (1)	103 (1)

a N, number of park users observed.

### Data collection

Data were collected by using SOPARC in each of the 24 parks during spring, summer, and autumn seasons observing during four 1-hour intervals on 2 weekdays and 2 weekend days each season (26). The hours were randomly chosen within segments of the day (morning, midday, afternoon, evening) covering daylight hours from 7 am to 8 pm. Each park was mapped and divided into discrete target areas (ie, a predetermined observation area in which park visitors could potentially engage in physical activity) and then observed systematically by trained observers who rotated through each area using momentary time sampling (ie, systematic and periodic scans were made of individuals and contextual factors). Target areas included outdoor facilities such as basketball courts, playing fields, picnic areas, and walkways, as well as indoor areas in recreation centers, such as gymnasiums, dance studios, and weight rooms.

Park neighborhoods were defined by a one-half-mile buffer around the park, about the distance of a typical walking trip (27). Because the population density of Chapel Hill is considerably lower than that of other cities, the block groups around the parks there were much larger. Only by including block groups with a centroid within four-fifths of a mile of the edge of each park was it possible to incorporate multiple block groups. Target areas in parks varied substantially in number and type, with a range of 23 to 33 areas in Chapel Hill, 17 to 30 in Albuquerque, 23 to 55 in Columbus, and 24 to 51 in Philadelphia. Target areas (N = 719) were visited a minimum of 16 times per season over 3 seasons, resulting in a total of 34,512 area visits (719 × 16 × 3) across all sites. During each visit, trained assessors first coded for the area characteristics (ie, accessible, usable, equipped, supervised, organized) and then completed systematic scans separately for females and males according to an established protocol (12). During a scan, the PA of each individual in a target area was coded as sedentary (ie, lying down, sitting, or standing), walking, or vigorous.

Observers were trained first by studying the operational definitions, instrument notation, and coding conventions. They then practiced and received feedback on their scoring of video examples from the SOPARC training digital video disc *(*DVD), followed by practice during live observations in diverse park settings. Certification was conferred if an observer met an accuracy rate of greater than 90% for the number of people counted and 85% for all other categories except race/ethnicity and challenging situations (eg, more than 5 people engaged in vigorous activity), where 80% reliability was accepted.

### Statistical analysis

We used the observation data to create 2 main outcomes of interest: 1) the number of park users and 2) the number and proportion of users engaging in MVPA per park per day. To assess whether the number of park users or the proportion of them engaging in MVPA varied by season or geographic region, we adopted multilevel models by using park day (7 am–8 pm) as the unit of analysis. Data for the 4 observations in each target area within a park were aggregated for each day of observation. For each park there were 12 repeated measures resulting from 4 observation days during each of 3 seasons. To model the 12 repeated measures nested within each park, we used generalized mixed-effect models. In these models, we treated parks as a random effect to allow for potential correlation of the 12 observation days nested within each park. The coefficients of both day-level and park-level covariates were treated as fixed effects. All models controlled for whether the day represented a weekend or weekday, season, and city. These last 2 covariates are the ones of primary interest in this study, so we investigated whether the effect of season varied by city by adding an interaction term between the 2 covariates. We modeled the number of park users with a Poisson regression and implemented robust standard errors to account for the overdispersion. The proportion of park users engaging in MVPA was modeled using a linear mixed-effect model.

## Results

Interobserver agreement, conducted on 15% of all observations by comparing the time-stamped data files on handheld personal digital assistants (PDAs), was high for all observations, with an overall mean of 94% (range, 85%–99%). Including only those visits during which a target area was occupied, the average agreement was 87% for the total number of people observed and 82% for race/ethnicity, 82% for age group, and 80% for PA level.

### Characteristics of park users

We observed 35,990 park users (Table 1a, Table 1b). Counts were similar for spring and autumn seasons (35% each); 30% of the total was observed during summer visits. Overall, approximately 55% of park users were male, ranging from 48% to 61% by city.

Overall, 50% were coded as white, 27% black, 13% Latino, and 10% other (eg, Asian, Native American), with substantial differences by city. Table 2a, Table 2b shows population estimates based on 2000 US Census block group data and the percentage of the population observed in sedentary, walking, and vigorous activity in the parks by season. In spite of using a larger neighborhood buffer, the largest number (Table 2a, Table 2b) and proportion of park users engaged in vigorous activities was in parks in Chapel Hill, and the largest proportion of park users engaged in sedentary activities was in Chapel Hill and Albuquerque, followed by Columbus and Philadelphia (Table 2a, Table 2b). Dividing the number of people using the park by the number of local residents indicated that, on average, fewer than 7% of the people living near the parks were observed in them during our assessment periods.

**Table 2a:** Characteristics of Observed Target Areas During 3 Seasons — Chapel Hill, North Carolina, and Albuquerque, New Mexico

City Average Frequency Population of Park Neighborhoods	Chapel Hill, North Carolina (35,666^a^)			Albuquerque, New Mexico (26,837^a^)		
**Seasons**	SP	SU	AU	SP	SU	AU
Accessible	97	96	98	99	98	98
Usable	99	99	100	99	97	98
Supervised	2	2	4	0	1	1
Equipped	2	2	2	0	2	1
Organized	2	1	2	0	0	0
Dark	0	0	1	0	1	4
Empty	71	77	69	69	75	77
**Proportion of park users**						
Population around the park (%)	13.1	8.5	15.4	11.5	7.2	6.6
Population in sedentary activity (%)	6.6	4.0	7.4	6.3	4.5	3.7
Population walking in park (%)	4.0	2.5	5.2	4.1	1.8	2.2
Population in vigorous activity (%)	2.6	1.9	2.9	1.1	0.8	0.7

a N, number of park users observed.

**Table 2b:** Characteristics of Observed Target Areas During 3 Seasons — Columbus, Ohio, and Philadelphia, Pennsylvania

City Average Frequency Population of Park Neighborhoods	Columbus, Ohio (45,191^a^)			Philadelphia, Pennsylvania (109,969^a^)		
**Seasons**	SP	SU	AU	SP	SU	AU
Accessible	82	89	85	87	89	89
Usable	85	96	91	94	96	96
Supervised	0	2	2	1	1	0
Equipped	3	4	4	1	1	1
Organized	0	1	1	1	0	2
Dark	9	0	5	4	3	4
Empty	90	88	89	83	82	86
**Proportion of park users^b^**						
Population around the park (%)	3.4	6.1	4.7	2.9	2.7	2.9
Population in sedentary activity (%)	1.8	3.7	2.8	1.7	1.7	1.9
Population walking in park (%)	1.0	1.7	1.2	0.5	0.5	0.6
Population in vigorous activity (%)	0.6	0.7	0.7	0.7	0.5	0.4

Abbreviations: SP, spring; SU, summer; AU, autumn. Population estimates of one-half mile or four-fifths mile were used to determine boundaries.

a N, area visits by park users.

b Population values are based on 2000 US Census SF3 block group data. Summed imputed observations and estimated population by city/season and percentage = number observed divided by estimated population.

### Park characteristics

Overall, most activity areas were accessible (eg, not locked or rented to a private party) to the public during nearly all observation visits in Chapel Hill and Albuquerque, and more than 80% of visits in Columbus and Philadelphia (Table 2a, Table 2b). Parks in Albuquerque and Chapel Hill had higher rates of accessibility, in part because they had fewer indoor facilities likely to be locked. Target areas were usable (eg, not excessively wet or roped off for repair) for physical activity nearly all the time, except in Columbus during spring, when areas were not usable during 14% of visits because of budget cuts and reduction in hours of some recreation centers. Target areas were frequently empty during visits (80% overall), ranging by season in Philadelphia 82% to 86% of the time, Columbus 88% to 90% of the time, and Albuquerque and Chapel Hill 69% to 77% of the time. Only a small proportion of the target areas observed had portable equipment or organized activities, regardless of season and city.

### Predicting park use and park-based physical activity

Season and city affected both MVPA in parks and the total number of park users (Table 3). When modeling the proportion of park users engaging in MVPA, we found marginal differences by both city and season. In particular, the proportion of active park users was lower during the summer compared with spring and in Albuquerque and Philadelphia compared with Chapel Hill (all *P* <.05). We did not find strong evidence for the interaction between the city and season indicators (data not shown). However, the coefficients for the interaction between Albuquerque and summer and of Columbus and summer were marginally significant, suggesting that in Albuquerque and Columbus the proportion of park users engaging in MVPA was lower during the summer.

**Table 3:** Factors Associated With the Proportion of Active Park Users

Variables	Model for Proportion of Users Engaged in MVPA per Park per Day	Model for Total Users
	β (SE)	Incident Rate Ratio
Intercept	0.53 (.04)^a^	−
**Season**		
Autumn	0.002 (.03)	1.02
Summer	−0.04 (.02)^b^	0.85
Spring	1 [Reference]	
**City**		
Albuquerque	−0.08 (.04)^b^	0.51^b^
Columbus	0.01 (.05)	0.49
Philadelphia	−0.09 (.05)^b^	0.69
Chapel Hill	1 [Reference]	
**Day of week**		
Weekend	0.003 (.02)	1.49^a^
Weekday	1 [Reference]	

Abbreviations: MVPA, moderate-to-vigorous physical activity; SE, standard error. Population estimates of one-half mile or four-fifths mile were used to determine boundaries.

a *P* <.001.

b *P* <.05.

The extent to which people used parks was significantly higher on weekend days than weekdays, and park use was lower in Albuquerque than in Chapel Hill (both *P* <.05). In separate models, we found a significant interaction between seasons and city. In particular, we found that the coefficient of the interaction term between the summer and Columbus indicators was positive and significant (*P* = .007), suggesting that the rate of visitation to Columbus parks during the summer was not as low as the simpler model specified.

## Discussion

Researchers have used SOPARC to collect data on park users and park characteristics previously, and given its ease of use in different populations, geographic settings, and seasons, our experience suggests it has value as a surveillance tool. The high interobserver agreement scores demonstrate that SOPARC can be reliably and effectively used across geographic locations, across seasons, and by different users. The strength of direct observation instruments, and of SOPARC in particular, is that they provide contextually rich data to both investigators and park personnel. Data on park use provides administrators with a benchmark to guide services and programs to optimally serve the local population. Seeing that some parks can serve more than 13% of the population, for example, can suggest to administrators that a park currently serving fewer than 3% of the population should be able to increase its reach. Researchers can also use SOPARC to increase the understanding of park features and management practices and to track trends in park use over time and assess the effects of interventions.

Overall, we observed that parks served no more than a maximum of 7% of the local residents during each week of observation, and by using the proportion observed being physically active, we estimate that a maximum of 3% of residents at any one time were actually engaged in MVPA in the park. The figure represents maximum because it assumes that each person observed was a different individual, when in reality, it is more likely that some people came to the park frequently and were observed more than once.

Although there was some seasonal variation in park use, it was marginally significant. This finding suggests that climate (19,25) may have less to do with park use than park factors themselves, such as programming and park features (13,14). Surveillance, therefore, could reasonably be spread throughout the year, reducing the need for the intensive week-long observations that we used. Additionally, park employees could possibly use the SOPARC methods as they make their regular rounds to different park areas, and the data collected through tracking park use could be helpful in guiding efforts to stimulate continued park-based physical activity.

Our analysis revealed marginal effects for season and city, both for total park users and the proportion engaged in MVPA. However, we found a strong effect for number visiting on weekends, suggesting that more people go to the parks on Saturday and Sunday. Weekend users and weekday users were similar in the intensity of their activity levels, however. Among the reasons for greater attendance and increased physical activity levels in some parks were the presence of additional and more varied facilities, a finding consistently reported in smaller-scale studies (13,14,18,19). Parks with recreation centers attracted more visitors than those that did not have them and provided more supervised programs, such as before and after school activities and summer sport programs.

It is worth noting that in Finland, the only developed country experiencing a trend in increased physical activity, increases appear to be related to leisure-time physical activity. Finland has invested heavily in public parks and recreational facilities, and 90% of Finns report using them (28). Therefore, parks seem to be both a worthy surveillance target for MVPA and a promising site for interventions.

SOPARC has several limitations as a surveillance tool. In its current form, it does not distinguish MVPA levels by age group or race/ethnicity. Some studies have modified the instrument to target specific factors (12); alternatively, additional scans of areas could be conducted to address this limitation. Other limitations of direct observation instruments are that they require specific training and that data collection can be time consuming (13). In this study, we used SOPARC during four 1-hour observations per day on 2 weekdays and 2 weekend days and entered the data onto PDAs (23). This procedure reduces data collection and management time.

This study contributes to the literature by establishing the geographic and seasonal generalizability of the SOPARC instrument as a surveillance and evaluation tool that is suitable for monitoring park use and management practices, as well as where and when physical activity occurs. As a validated instrument, SOPARC can be used to collect data showing the relationships between park characteristics and levels of physical activity, setting the stage for interventions to attract more people and increase their activity levels in parks.
